# Regulation of Hippocampal Gamma Oscillations by Modulation of Intrinsic Neuronal Excitability

**DOI:** 10.3389/fncir.2021.778022

**Published:** 2022-01-26

**Authors:** Alexander Klemz, Florian Wildner, Ecem Tütüncü, Zoltan Gerevich

**Affiliations:** Institute of Neurophysiology, Charité—Universitätsmedizin, Berlin, Germany

**Keywords:** SK channel, BK channel, IK channel, KCNQ2, KCNQ3, Cav3, Cav3.2, Cav3.3

## Abstract

Ion channels activated around the subthreshold membrane potential determine the likelihood of neuronal firing in response to synaptic inputs, a process described as intrinsic neuronal excitability. Long-term plasticity of chemical synaptic transmission is traditionally considered the main cellular mechanism of information storage in the brain; however, voltage- and calcium-activated channels modulating the inputs or outputs of neurons are also subjects of plastic changes and play a major role in learning and memory formation. Gamma oscillations are associated with numerous higher cognitive functions such as learning and memory, but our knowledge of their dependence on intrinsic plasticity is by far limited. Here we investigated the roles of potassium and calcium channels activated at near subthreshold membrane potentials in cholinergically induced persistent gamma oscillations measured in the CA3 area of rat hippocampal slices. Among potassium channels, which are responsible for the afterhyperpolarization in CA3 pyramidal cells, we found that blockers of SK (K_Ca_2) and K_V_7.2/7.3 (KCNQ2/3), but not the BK (K_Ca_1.1) and IK (K_Ca_3.1) channels, increased the power of gamma oscillations. On the contrary, activators of these channels had an attenuating effect without affecting the frequency. Pharmacological blockade of the low voltage-activated T-type calcium channels (Ca_V_3.1–3.3) reduced gamma power and increased the oscillation peak frequency. Enhancement of these channels also inhibited the peak power without altering the frequency of the oscillations. The presented data suggest that voltage- and calcium-activated ion channels involved in intrinsic excitability strongly regulate the power of hippocampal gamma oscillations. Targeting these channels could represent a valuable pharmacological strategy against cognitive impairment.

## Introduction

The intrinsic excitability of a neuron describes the probability of action potential firing in response to synaptic inputs (Dunn and Kaczorowski, 2019). The magnitude of excitability is plastic and under ongoing modulation by voltage- and calcium-activated ion channels located directly at the input or output side of neurons, a phenomenon called intrinsic plasticity (Kourrich et al., 2015; Debanne et al., 2019). Alongside its better-known counterpart synaptic plasticity, intrinsic plasticity is thought to play a major role in information processing, learning, and memory (Lisman et al., 2018). Reduction in baseline intrinsic excitability or disturbance in intrinsic plasticity has been linked to cognitive deficits in both normal aging and neuropsychiatric disorders such as Alzheimer’s disease (Kaczorowski and Disterhoft, 2009; Eslamizade et al., 2015).

Several subthreshold voltage and calcium-activated channels are expressed in hippocampal neurons, open below the threshold of action potentials at around −55 mV and are thus able to modulate intrinsic excitability in hippocampal networks. Potassium channels activated by increases in the intracellular calcium concentration (K_Ca_) have been shown to effectively modulate the firing patterns of neurons (King et al., 2015). Among them, the firstly described big-conductance K_Ca_ channel (BK, Slo1, or K_Ca_1.1) is the only one that is also activated by voltage (Almássy and Nánási, 2019). It is expressed in the brain and contributes mainly to the repolarization phase of action potentials and the fast component of afterhyperpolarization (AHP; Contet et al., 2016). Three subtypes of the small conductance K_Ca_ channels (SK1-3 or K_Ca_2.1-3) and one intermediate-conductance K_Ca_ (IK or K_Ca_3.1) are known (Adelman et al., 2012). They are all voltage-independent, gated directly by submicromolar concentrations of intracellular Ca^2+^, and rapidly modulate the intrinsic excitability of neurons mainly by generating the slower components of the AHP (Bond et al., 2005; Pedarzani and Stocker, 2008). Blockade of K_Ca_2 channels with apamine has been shown to improve hippocampus-dependent learning in rodents (Deschaux et al., 1997; Stackman et al., 2002).

The voltage-activated Kv7 channels (KCNQ) also open at near resting membrane potential. Their slow gating kinetics, missing inactivation, and location at input and output sites of neurons make them ideal for controlling the intrinsic excitability and the output of neurons (Greene and Hoshi, 2017). The current through the Kv7.2/3 subtypes (KCNQ2/3) was originally described as the M-current because the channel is coupled to the muscarinic M1 and M3 receptors and activation of these Gq coupled receptors suppresses the current increasing neuronal excitability (Brown and Adams, 1980). Other Gq coupled receptors are also able to block KCNQ2/3 channels and depolarize the resting membrane potential, lower the threshold of action potentials and increase predominantly the slow component of the AHP (Shapiro, 2000; Delmas and Brown, 2005). Blockade of the KCNQ2/3 channels by ligands such as linopirdine or XE991 has been shown to have pro-cognitive/memory-enhancing effects in both animal models and humans (Chorvat et al., 1998; Gribkoff, 2003; Baculis et al., 2020).

T-type calcium channels (Cav3.1-3) are the only voltage-gated Ca^2+^ channels that are activated below the threshold (Weiss and Zamponi, 2019). They are expressed on both the dendrites and axon initial segments and are able to impact the intrinsic excitability of neurons efficiently. Loss-of-function mutations of T-type channels have been linked to autism spectrum disorder (Splawski et al., 2006), and schizophrenia (Andrade et al., 2016), and activation of the channel was shown to enhance long-term potentiation in cortical slices (Moriguchi et al., 2012) and memory-related behavior (Gangarossa et al., 2014; Yabuki et al., 2017; Fukunaga et al., 2019; Degawa et al., 2021; Yuan et al., 2021).

Gamma oscillations are rhythmic fluctuations of neuronal activity at frequencies from 30 to 90 Hz generated by a circuit containing feedback inhibitory inputs predominantly from parvalbumin-positive fast-spiking inhibitory interneurons providing perisomatic inhibition onto pyramidal cells (Bartos et al., 2001; Buzsáki and Wang, 2012). They are thought to play a key role in higher cognitive functions by supplying the background of information processing within and between brain areas (Womelsdorf and Fries, 2006) and their disturbances have been observed in a wide range of neuropsychiatric diseases with cognitive symptoms (Mably and Colgin, 2018), such as schizophrenia (Kwon et al., 1999; Hunt et al., 2017; Lemercier et al., 2017), autism (Grice et al., 2001; Casanova et al., 2020; Kayarian et al., 2020) and Alzheimer’s disease (Ribary et al., 1991; Arroyo-García et al., 2021). The gamma oscillation generating circuit is connected by excitatory and inhibitory synapses; however, the firing and therefore synaptic output of the cells depend on the intrinsic excitability of the neurons involved in the circuit. While the synaptic components of the circuits and their modulation of gamma oscillations have been widely investigated in the last decades, the role of intrinsic plasticity remained much less examined. The goal of the present work was to explore which subthreshold activated ion channels, involved in intrinsic plasticity, are able to modulate cholinergically induced gamma oscillations in the hippocampus. Hyperpolarization-activated cyclic nucleotide-gated (HCN) channels were excluded from the study because their effect on gamma oscillations was intensively investigated in previous works (Fisahn et al., 2002, 2004; Boehlen et al., 2009; Pietersen et al., 2009; Neymotin et al., 2013).

## Methods and Materials

### Animals and Slice Preparation

All animal procedures were conducted in accordance with the guidelines of the European Communities Council and the institutional guidelines approved by the Berlin Animal Ethics Committee (Landesamt für Gesundheit und Soziales Berlin, T0330/12). All studies involving animals are reported in accordance with the ARRIVE guidelines (du Sert et al., 2020).

For local field potential (LFP) recordings, Wistar rats of both sexes at an age of 5–9 weeks were anesthetized with isoflurane and then decapitated as described earlier (Schulz et al., 2012a; Lemercier et al., 2015). Their brains were quickly removed from the skull and submerged in ice-cold carbogenated (95% O_2_, 5% CO_2_) sucrose-based solution with an osmolality of ~330 mOsm/kg of the following composition (in mM): NaCl, 80; NaHCO_3_, 25; NaH_2_PO_4_, 1.25; KCl, 2.5; glucose, 25; sucrose, 85; CaCl_2_, 0.5; MgCl, 3. Hippocampal slices were prepared by cutting the brain into 400 μm thick horizontal slices at an angle of 13° in fronto-occipital direction with a DTK-1000 vibratome (DSK, Dosaka, Japan) and immediately transferred to an interface-type recording chamber perfused with carbogenated, warm (32–34°C) artificial cerebrospinal fluid (ACSF; flow rate of 1.6–1.7 ml/min; osmolality of ~300 mOsm/kg) containing (in mM): NaCl, 129; NaH_2_PO_4_, 1.25; NaHCO_3_, 21; glucose, 10; MgSO_4_, 1.8; CaCl_2_, 1.6; KCl, 3. Slices were left for recovery for at least 1 h before the beginning of recordings.

For patch clamp experiments, acute brain slices were obtained from 20- to 26-day-old male Wistar rats. Horizontal hippocampal slices (300 μm thick) were cut using a vibratome (Leica VT 1200, Leica Biosystems, Wetzlar, Germany). After preparation, slices were stored at 34°C in submerged condition for at least 30 min for recovery and kept at room temperature afterwards. The same sucrose-based solution as described above was used for slicing and storage.

### Extracellular Recordings and Analysis

Measurement electrodes were put into glass pipettes filled with ACSF and then placed in the stratum pyramidale in CA3b of the hippocampus. LFPs were low-pass filtered at 1 kHz and digitized by a CED 1401 interface (Cambridge Electronic Design, Cambridge, UK) at 5 kHz (Klemz et al., 2021). Gamma oscillations were induced by bath application of acetylcholine (ACh, 10 μM) and the acetylcholine esterase inhibitor physostigmine (Phys; 2 μM; Schulz et al., 2012b). After stabilization of gamma oscillations, drugs were administered 100 min after induction for 60 min. Since induced gamma oscillations are not stable during long application times, time-matched control experiments without drug applications were carried out simultaneously with slices from the same animals.

Analysis of extracellular recordings was carried out by calculating power spectra every 2 min with a 120 s window throughout the whole recording (Meier et al., 2020). Peak power and peak frequency of the oscillations were extracted using a custom-made script for the Spike2 software (Cambridge Electronic Design, Cambridge, UK). Network activity was considered a gamma oscillation when the power spectrum had a peak between 30 and 80 Hz and the Q factor (peak frequency/half bandwidth) of the oscillation was supercritical (>0.5; Lemercier et al., 2017). Peak power and peak frequency were normalized to a 10-min window after 90 min of induction where oscillations were already stabilized. This period corresponds to the time immediately before drug application (90–100 min) or the time-matched period in control experiments. Normalized peak power and peak frequency of a 10-min window 60 min after drug application (150–160 min) were compared to time-matched control of the same time window. Data are presented as mean ± SEM. Statistical comparison was made using Student’s *t*-test or ANOVA with a *post-hoc* test. The baseline activity for XE991 experiments was analyzed by creating power spectra of 10 min before the induction and after 160 min of bath application of XE991 and calculating the mean power (integral power divided by the number of bins) between 10 and 50 Hz. Since the power of LFP signals is log-normally distributed, mean power data are presented as geometric mean and statistically analyzed by Student’s *t*-test of the logarithms. The significance level was set at *p* < 0.05.

### Patch Clamp Recordings and Analysis

After the recovery period, slices were individually transferred to a submerged type recording chamber and continuously perfused at a flow rate of ~5 ml/min with carbogenated ACSF containing (in mM): NaCl, 95; TEA-Cl, 25; NaHCO_3_, 25; NaH_2_PO_4_, 1.25; KCl, 2.5; CaCl_2_, 2; MgCl_2_, 1; Glucose, 25 (osmolality 310 ± 5 mOsm/kg) at room temperature (22–24°C). For detection of calcium currents, tetrodotoxin (0.5 μM), picrotoxin (100 μM), 6-cyano-7-nitroquinoxaline-2,3-dione (CNQX, 10 μM), and D-(−)-2-amino-5-phosphonopentanoic acid (D-APV, 50 μM) were added to the external solution to block voltage-gated sodium channels, GABA_A_ receptors, AMPA receptors, and NMDA receptors, respectively. Whole-cell patch clamp recordings were performed on CA1 pyramidal cells in voltage clamp mode. CA1 pyramidal cells were used for these experiments as a model, since they are less active spontaneously and express T-type channels similar to CA3 pyramidal cells (McKay et al., 2006). The developmental expression of the channels in the hippocampus peaks at around P21 and then declines at P60 (Aguado et al., 2016), enabling the investigation of younger animals in the patch clamp recordings. CA1 neurons were identified visually using infrared differential interference contrast microscopy of a Zeiss Axioskop (Carl Zeiss AG, Oberkochen, Germany). Patch electrodes were pulled from borosilicate glass capillaries (1.5 mm outer/0.86 mm inner diameter; Science Products, Hofheim, Germany) and had a resistance of 3–6 MΩ when filled with internal solution containing (in mM): CsCl, 115; TEA-Cl, 20; HEPES, 10; EGTA, 10; MgCl_2_, 2; Na_2_-ATP, 2; Na_2_-GTP, 0.5; Na_2_-phosphocreatin, 5; pH adjusted to 7.2 using CsOH, osmolality ~300 mOsm/kg. Recordings were obtained using an EPC9 amplifier (HEKA, Heidelberg, Germany). Signals were low-pass filtered at 2.9 kHz using the amplifier’s built-in 4-pole low-pass Bessel filter and digitized at 10 kHz. Series resistance was not corrected but monitored by application of a brief voltage step of −10 mV and recordings were discarded if they changed by more than 30%. Whole-cell capacitance was compensated during these recordings using the amplifier’s automatic capacitance transient cancellation circuitry. Liquid junction potential was not corrected. PatchMaster and FitMaster software (HEKA, Heidelberg, Germany) were used for acquisition and analysis, respectively. To evoke voltage-gated calcium currents, cells were held at a membrane potential of −80 mV, and steps to depolarized voltages were applied. To separate low voltage-activated from high voltage-activated calcium currents, a two-step protocol with depolarizing voltage steps from −80 to −40 and subsequently from −40 to −10 mV was applied. Data were leak corrected by a P/4-protocol as described earlier (Chad and Eckert, 1986; Büsselberg et al., 1992). Values are given as mean ± SEM. If not stated differently, the significance of differences before and after drug application was assessed using paired Student’s *t-*test. The differences were considered significant when *p* < 0.05.

### Drugs

Physostigmine (Phys), NS309, NS6180, UCL1684, NS19504, Penitrem A, XE991, ICA110381, SAK3, and NNC55-0396, CNQX, D-APV, and TTX were obtained from Tocris Bioscience (Bristol, UK). Acetylcholine (ACh) was purchased from Sigma-Aldrich (Taufkirchen, Germany), TTA-P2 from Alomone Labs (Jerusalem, Israel), picrotoxin from Abcam (Cambridge, UK). Drugs were dissolved in water or DMSO and further diluted with ACSF to achieve the respective drug concentrations. Final DMSO concentration was held below 0.02% or DMSO control experiments were carried out. We did not investigate the effect of DMSO on gamma oscillation power systematically; however, it is known that DMSO reduces neuronal excitability (Tamagnini et al., 2014), which can explain the lower normalized DMSO control values. In patch clamp experiments, the final DMSO concentration was 0.2%.

## Results

### Activation of K_Ca_2 Channels Inhibits and Blockade Increases Gamma Oscillations

**Figure 1:**
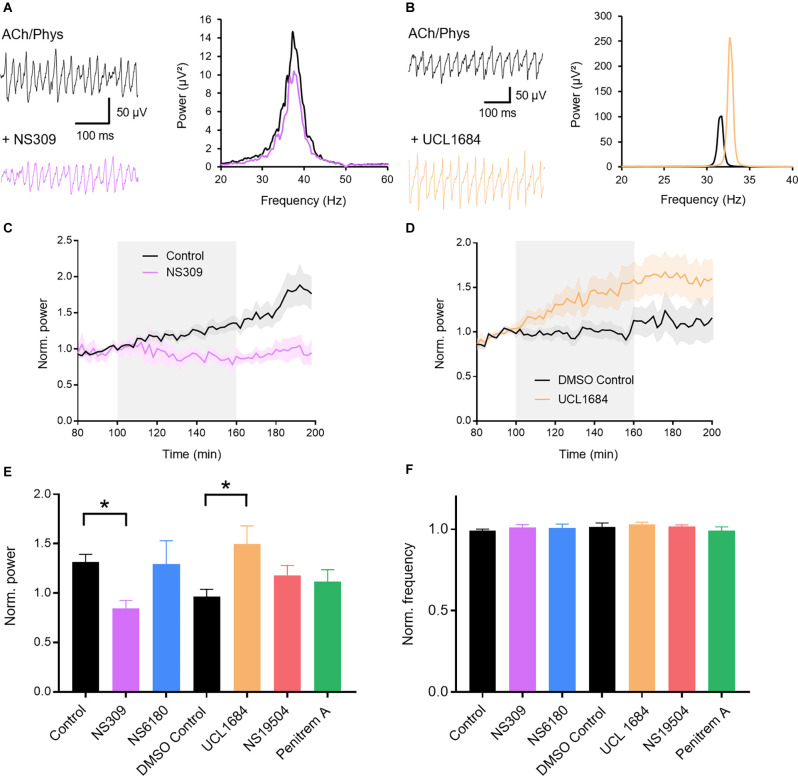
Role of calcium-activated potassium channels (K_Ca_) in hippocampal gamma oscillations. **(A)** Original traces (left) and power spectra (right) of cholinergic gamma oscillations before (black) and after (purple) bath application of the K_Ca_2 and K_Ca_3.1 activator NS309 (3 μM). **(B)** Original traces (left) and power spectra (right) of cholinergic gamma oscillations before (black) and after (orange) bath application of the K_Ca_2 antagonist UCL1684 (0.1 μM). **(C)** Normalized gamma power before, during and after the application of NS309 (3 μM, gray area) in comparison to control. **(D)** Normalized gamma power over time around the application of UCL 1684 (0.1 μM, gray area) in comparison to control. **(E)** Effect of K_Ca_ activators and blockers on the normalized peak power of hippocampal gamma oscillations compared to time matched and solvent controls. Control: *n* = 13 slices, *N* = 9 animals; NS309 (3 μM): *n* = 6, *N* = 3, *p* = 0.042; NS6180 (1 μM): *n* = 7, *N* = 3, *p* = 0.990; DMSO control: *n* = 8, *N* = 8; UCL1684 (0.1 μM): *n* = 12, *N* = 6, *p* = 0.032; NS19504 (10 μM): *n* = 14, *N* = 6, *p* = 0.565; Penitrem A (0.2 μM): *n* = 10, *N* = 5, *p* = 0.814. See selectivity in “Results” section. **(F)** Effect of K_Ca_ activators and blockers on the normalized peak frequency of hippocampal gamma oscillations compared to time matched and solvent controls. NS309 (3 μM): *p* = 0.664; NS6180 (1 μM): *p* = 0.760; UCL1684 (0.1 μM): *p* = 0.858, NS19504 (10 μM): *p* = 0.988; Penitrem A (0.2 μM): *p* = 0.767. Recording temperature was between 32 and 34°C. Traces were lowpass filtered at 200 Hz and bandstop filtered at 50 Hz. Bars show mean ± SEM. *p < 0.05. ACh, acetylcholine; Phys, physostigmine.

We first investigated whether pharmacological manipulation of K_Ca_ channels can modulate gamma oscillations. Gamma oscillations were induced by bath application of acetylcholine (10 μM) and the acetylcholine esterase inhibitor physostigmine (2 μM) in the CA3 region of rat hippocampal slices. Peak power and frequency stabilized after about 80 min, enabling time-controlled pharmacological testing between slices. We applied activators and blockers of the different K_Ca_-subtypes after 100 min of induction. The less selective activator of K_Ca_2 and K_Ca_3.1, NS309 (3 μM; 0.848 ± 0.078, *n* = 6), significantly decreased normalized gamma power compared to control (1.315 ± 0.077, *n* = 13; *p* = 0.0421; Figures 1A,C,E) suggesting that K_Ca_ channels are involved in hippocampal gamma oscillations. The peak frequency of the oscillations stayed unaffected (control: 0.992 ± 0.009; NS309: 1.014 ± 0.011; *p* = 0.664; Figures 1A,F). We then used more selective drugs to investigate the role of different K_Ca_ subtypes in more detail. NS6180 (1 μM), an antagonist of K_Ca_3.1 channels, had no significant effect on either peak gamma power (control: 1.315 ± 0.077, *n* = 13; NS6180: 1.293 ± 0.237, *n* = 7, *p* = 0.990; Figure 1E) nor frequency (control: 0.992 ± 0.009; NS6180: 1.007 ± 0.025; *p* = 0.760; Figure 1F). In contrast, the K_Ca_2 channel antagonist UCL1684 (0.1 μM) significantly increased peak gamma power (1.181 ± 0.100, *n* = 12; DMSO control: 0.966 ± 0.072, *n* = 8; *p* = 0.032; Figures 1B,D,E) without altering the frequency of the oscillations (1.030 ± 0.015; DMSO control: 1.014 ± 0.026; *p* = 0.858; Figures 1B,F), suggesting that K_Ca_2 and not K_Ca_3.1 channels are involved in the maintenance of cholinergic gamma oscillations. Finally, to complete our investigation of K_Ca_ we applied the K_Ca_1.1 activator NS19504 (10 μM) and the K_Ca_1.1 blocker Penitrem A (0.2 μM). Both ligands left gamma oscillations unaffected (power: DMSO control: 0.966 ± 0.072, *n* = 8; NS19504: 1.181 ± 0.100, *n* = 14, *p* = 0.565; Penitrem A: 1.117 ± 0.121, *n* = 10, *p* = 0.814; Figure 1E; frequency: DMSO control: 1.014 ± 0.026; NS19504: 1.020 ± 0.008, *p* = 0.988; Penitrem A: 0.992 ± 0.023, *p* = 0.767; Figure 1F). In conclusion, these results indicate that only K_Ca_2 channels exert an influence over cholinergically induced gamma oscillations in the rat hippocampus. K_Ca_2 activation decreases while blockade increases gamma oscillation power.

### KCNQ2/3 Channel Activation Inhibits and Blockade Increases Gamma Oscillations

**Figure 2:**
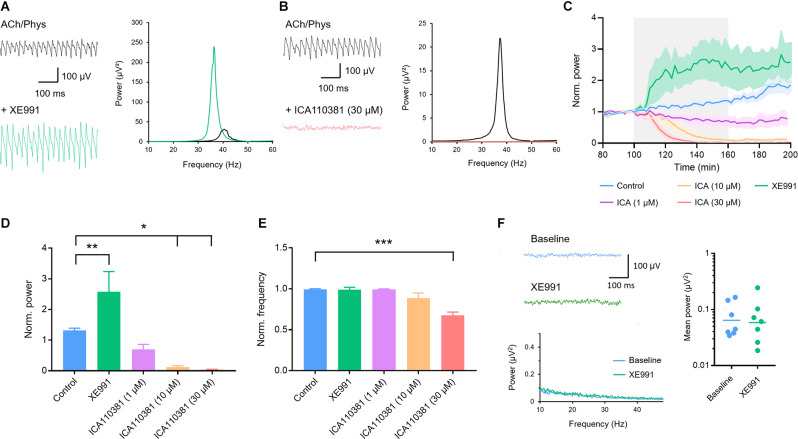
Involvement of voltage-gated Kv7.2/3 (KCNQ2/3) channels in the modulation of hippocampal gamma oscillations. **(A)** Original traces (left) and power spectra (right) of cholinergically induced gamma oscillations before (black) and after (green) bath application of the KCNQ2/3 blocker XE991 (10 μM). **(B)** Original traces (left) and power spectra (right) of cholinergically induced gamma oscillations before (black) and after (red) bath application of the KCNQ2/3 positive allosteric modulator ICA110381 (30 μM). **(C)** Normalized gamma power before, during and after the application of XE991 (10 μM, green) and increasing concentrations of ICA110381 (1 μM; purple; 10 μM; orange; 30 μM red) compared to control (blue). The application of the drugs is highlighted in gray. **(D)** Effect of XE991 and ICA110381 on the normalized peak power of hippocampal gamma oscillations compared to control. Control (blue): *n* = 13 slices, *N* = 9 animals; XE991 (green): *n* = 9, *N* = 4, *p* = 0.009; ICA110381 1 μM: (purple): *n* = 8, *N* = 5, *p* = 0.381; 10 μM (orange): *n* = 8, *N* = 4, *p* = 0.019; 30 μM (red): *n* = 8, *N* = 4, *p* = 0.011. **(E)** Effect of XE991 and ICA110381 on the normalized peak frequency of hippocampal gamma oscillations compared to control. XE991 (green): *p* > 0.999; ICA110381 1 μM (purple): *p* > 0.999; 10 μM (orange): *p* = 0.073; 30 μM (red): *p* < 0.001. **(F)** Left, top: original traces of local field potentials (LFPs) before and after the application of XE991. Left, bottom: power spectra of LFPs before and after XE991 application. Right: scatter plot comparing the mean absolute power between 10 and 50 Hz before (baseline; blue) and after application of XE991 (green, *n* = 7, *p* = 0.825, bars show geometric mean). Recording temperature was between 34 and 36°C. Traces were lowpass filtered at 200 Hz and bandstop-filtered at 50 Hz. Bars show mean ± SEM. *p < 0.05, **p < 0.01, ***p < 0.001. ACh, acetylcholine; Phys, physostigmine.

KCNQ2/3 (Kv7.2/3) channels are widely distributed in the hippocampus (Klinger et al., 2011) where they contribute to the control of neuronal excitability (Greene and Hoshi, 2017; Carver and Shapiro, 2019). Application of the KCNQ2/3 blocker XE991 (10 μM) strongly increased the peak power of fully developed oscillations (control: 1.315 ± 0.077, *n* = 13; XE991: 2.581 ± 0.658, *n* = 9; *p* = 0.009; Figures 2A,C,D) while the frequency did not change (control: 0.992 ± 0.009; XE991: 0.990 ± 0.030; *p* > 0.999; Figures 2A,E). ICA110381, a positive allosteric modulator of KCNQ2/3, applied at 30 μM harshly inhibited gamma oscillations and completely abolished them in six out of eight slices (0.039 ± 0.012; control: 1.315 ± 0.077, *n* = 13; *p* < 0.001; Figures 2B–D), which made the evaluation of the peak frequency unreliable (0.678 ± 0.0384; control: 0.992 ± 0.009; Figures 2B,E). Lower concentrations (10 and 1 μM) concentration-dependently decreased gamma power (control: 1.315 ± 0.077, *n* = 13; 10 μM: 0.121 ± 0.0481, *n* = 8, *p* = 0.019; 1 μM: 0.704 ± 0.156, *n* = 8, *p* > 0.381; Figures 2C,D) while the frequency was unchanged (control: 0.992 ± 0.009; 10 μM: 0.886 ± 0.063, *p* = 0.073; 1 μM: 0.995 ± 0.004, *p* > 0.999; Figure 2E). Since these results indicate that cholinergically induced gamma oscillations are highly dependent on KCNQ2/3 channels, we next tried to induce neuronal oscillations with XE991 (10 μM) in CA3. Bath application of the KCNQ2/3 blocker for up to 160 min neither induced gamma oscillations nor increased the baseline activity in the low gamma frequency range (baseline integral power: geometric mean: 0.064 μV^2^; XE991: geometric mean: 0.058 μV^2^, *n* = 7, *p* = 0.825; *p* = 0.825; Figure 2F) demonstrating that blockade of KCNQ2/3 channels alone is not sufficient to evoke synchronized oscillatory activity.

### T-Type Calcium Channels Are Involved in Hippocampal Gamma Oscillations

**Figure 3:**
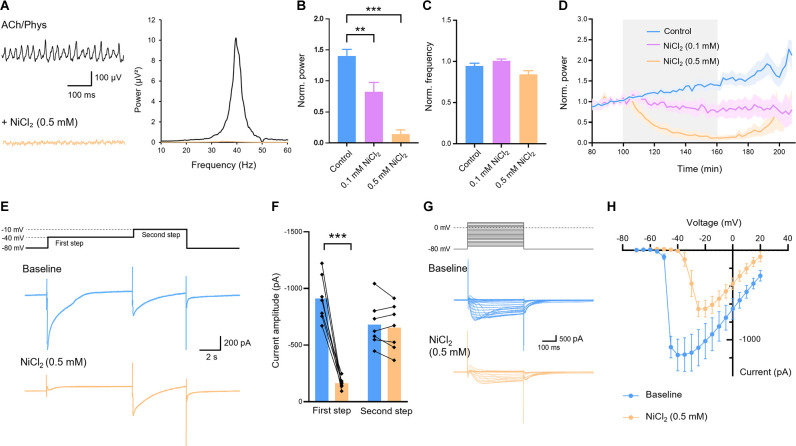
Effect of T-Type calcium channel blocker nickel chloride (NiCl_2_) on cholinergic gamma oscillations in the rat hippocampus. **(A)** Original traces (left) and power spectrum (right) of hippocampal gamma oscillations before (black) and after (orange) application of NiCl_2_ (0.5 mM). Local field potential traces were lowpass filtered at 200 Hz and bandstop filtered at 50 Hz. **(B)** Normalized power of cholinergically induced gamma oscillations after bath application of NiCl_2_ compared to control. Control (blue): *n* = 13 slices, *N* = 9 animals; NiCl_2_ 0.1 mM (purple): *n* = 7, *N* = 3, *p* = 0.002; 0.5 mM (orange): *n* = 11, *N* = 4, *p* < 0.001. **(C)** Normalized frequency after application of NiCl_2_ compared to control. NiCl_2_0.1 mM (purple): *p* = 0.406; 0.5 mM (orange): *p* = 0.088. **(D)** Normalized power before, during and after bath application (gray) of different concentrations of NiCl_2_ (0.1 mM, purple; 0.5 mM orange) compared to control (blue). **(E)** Low voltage- and high voltage-activated calcium currents recorded by a first depolarizing step from −80 to −40 mV and a second step from −40 to −10 mV, respectively, of the same cell before (blue) and after (orange) bath application of NiCl_2_ (0.5 mM). **(F)** Amplitudes of low and high voltage-activated calcium currents evoked by the first and second step, respectively, before and after application of NiCl_2_ (0.5 mM). First step: *p* < 0.001. Second step: *p* = 0.331; *n* = 7, *N* = 4, paired *t*-test. **(G)** Step protocol (above) and original traces of calcium currents (below) with voltage steps from −70 mV to +20 mV and holding potential of −80 mV before (blue) and after (orange) bath application of NiCl_2_ (0.5 mM). **(H)** Amplitudes of calcium currents evoked by depolarizing steps as described in **(G)**. For LFP experiments recording temperature was between 34 and 36°C. Patch-clamp experiments were carried out at room temperature (22–24°C). Data shown as mean ± SEM. **p < 0.01, ***p < 0.001. ACh, acetylcholine; Phys, physostigmine.

Next we investigated the role of T-type calcium channels for cholinergic gamma oscillations in hippocampal slices. We applied NiCl_2_, a blocker of T-type calcium channels, at a concentration of 0.5 mM which strongly reduced gamma power and in 8 out of 11 slices completely abolished it (0.138 ± 0.070; control: 1.401 ± 0.107, *n* = 13; *p* < 0.001; Figures 3A,B,D). The frequency decreased, however, the complete abolishment of oscillations in the majority of slices made the evaluation of peak frequency uncertain (0.845 ± 0.044; control: 0.946 ± 0.034; *p* = 0.088; Figures 3A,C). Therefore, we tested a lower concentration of 0.1 mM NiCl_2_ and found a significant inhibition of gamma power (0.824 ± 0.149, *n* = 7; control: 1.401 ± 0.107, *n* = 13; *p* = 0.002; Figures 3B,D) with no significant alterations in oscillation frequency (1.002 ± 0.021; control: 0.946 ± 0.034; *p* = 0.406; Figure 3C).

Next, we asked whether the applied concentration of 0.5 mM NiCl_2_ was selective for the T-type channels or other voltage-activated calcium channels were also blocked. Therefore, we evoked low voltage-activated calcium currents in CA1 pyramidal cells by voltage steps from −80 to −40 mV (Figure 3E) which correspond to the currents *via* T-type channels (Figure 3E). NiCl_2_ (0.5 mM) significantly reduced these currents (910.9 ± 75.9 pA to 162.2 ± 17.89 pA, *p* < 0.001) without affecting the high voltage-activated currents evoked by a subsequent voltage step from −40 to −10 mV (682.3 ± 74.69 pA to 652.7 ± 76.52 pA, *p* = 0.331; Figures 3E–H). These results confirm that NiCl_2_ at the applied concentration that inhibited gamma oscillations selectively blocked T-type calcium channels.

**Figure 4:**
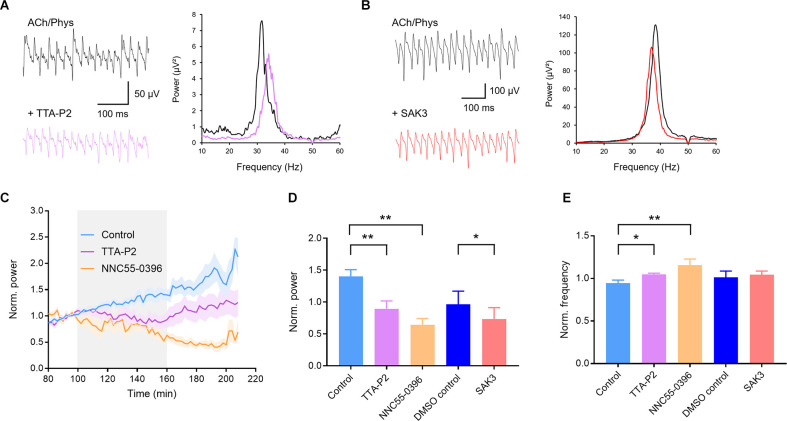
Selective ligands of T-type calcium channels inhibit cholinergic gamma oscillations in rat hippocampal slices. **(A)** Original traces (left) and power spectra (right) of cholinergic gamma oscillations before (black) and after (purple) application of the selective T-type calcium channel blocker TTA-P2 (1 μM). **(B)** Original traces (left) and power spectra (right) of cholinergic gamma oscillations before (black) and after (red) application of the selective T-type calcium channel enhancer SAK3 (0.1 μM). **(C)** Normalized power before, during and after bath application (gray) of the selective T-type blocker TTA-P2 (1 μM, purple) and NNC55-0396 (100 μM, orange) compared to control (blue). **(D)** Effect of the T-type calcium channel blockers TTA-P2 and NNC55-0396 as well as the enhancer SAK3 on the power of hippocampal gamma oscillations compared to time matched and solvent control. Control (light blue): *n* = 13 slices, *N* = 9 animals; TTA-P2 (1 μM, purple): *n* = 14, *N* = 6, *p* = 0.005, NNC55-0396 (100 μM, orange): *n* = 6, *N* = 2, *p* = 0.001; DMSO control (dark blue): *n* = 8, *N* = 7; SAK3 (0.1 μM, red): *n* = 10, *N* = 6, *p* = 0.020.** (E)** Effect of the T-type calcium channel blockers TTA-P2 and NNC55-0396 as well as the enhancer SAK3 on the peak frequency of hippocampal gamma oscillations compared to time matched and solvent control. TTA-P2 (1 μM, purple): *p* = 0.042; NNC55-0396 (100 μM, orange): *p* = 0.001; SAK3 (0.1 μM, red): *p* = 0.306. Recording temperature was between 34 and 36°C. Traces were lowpass filtered at 200 Hz and bandstop filtered at 50 Hz. Bars show mean ± SEM. *p < 0.05, **p < 0.01. ACh, acetylcholine; Phys, physostigmine.

Further utilization of selective T-type channel ligands confirmed our findings with NiCl_2_. The selective T-type blockers TTA-P2 (1 μM) and NNC55-0396 (100 μM) both significantly reduced gamma power (control: 1.401 ± 0.107, *n* = 13; TTA-P2: 0.895 ± 0.123, *n* = 14; *p* = 0.005; Figures 4A,C,D; NNC55-0396: 0.645 ± 0.096, *n* = 6, *p* = 0.001; Figures 4C,D) and increased oscillation frequency (control: 0.946 ± 0.034; TTA-P2: 1.049 ± 0.013, *p* = 0.042; NNC55-0396: 1.156 ± 0.072, *p* = 0.001; Figure 4E). The T-type activator SAK3 (0.1 μM) also significantly decreased gamma power (0.733 ± 0.057, *n* = 10, DMSO control: 0.966 ± 0.072, *n* = 8; *p* = 0.020; Figures 4B,D) with no effect on oscillation frequency (1.043 ± 0.014; DMSO control: 1.014 ± 0.026; *p* = 0.306; Figures 4B,E). In conclusion, these results suggest that subthreshold activated T-Type calcium channels regulate cholinergic gamma oscillations.

## Discussion

The present study was carried out to investigate which subthreshold activated channels are able to modulate gamma oscillations in the hippocampal CA3 area. These channels open by membrane voltage change below or around the threshold of action potentials or by an increase of intracellular calcium concentration and are important regulators of intrinsic neuronal excitability. Our results show for the first time that among these subthreshold channels, K_Ca_2, Kv7.2/3, as well as T-type calcium channels can modulate the power and, in some cases, also the peak frequency of hippocampal gamma oscillations and herewith changes in neuronal networks. Since changes in gamma oscillation power may provide a disease biomarker to investigate novel therapies against cognitive impairment in diseases (Honda et al., 2020; Meier et al., 2020), the present data suggest that K_Ca_2, KCNQ2/3, and T-type calcium channel modulators might be promising targets in these states.

Since the postulate of Donald Hebb (Hebb, 1949) and the later discovery of long-term potentiation of synaptic transmission (Bliss and Lømo, 1973), activity-dependent changes in the strength of synapses were primarily thought to mediate memory storage in the brain. Therefore, investigation of synapses and the modulation of synaptic strength have dominated neuroscience research in the last decades. Accumulating evidence suggests, however, that synaptic plasticity is not the only neuronal change leading to the storage of information but global cellular properties such as intrinsic excitability of neurons and its plastic changes also play an important role in learning and memory (Disterhoft et al., 1986; Lisman et al., 2018). The plasticity of intrinsic excitability is like synaptic plasticity bi-directional and displays cell and input specificity (Debanne et al., 2019). It is mediated by ion channels located in dendrites, soma, and axon of the neuron able to modulate postsynaptic potentials, resting membrane potential, and action potential threshold, respectively.

The ion channels involved in these processes are heterogenous and predominantly activated by subthreshold voltage changes or increases in intracellular calcium concentration. Here, we investigated ion channels that are classically involved in intrinsic excitability in the CA3 area of the hippocampus. Increasing the intrinsic excitability by opening T-type channels or blocking potassium channels might increase the gamma power by reducing accommodation, enabling neurons to fire on more waves of an oscillation and promoting action potentials after perisomatic IPSPs (Traub et al., 2004). The power of *in vitro* gamma oscillations was shown to correlate well with *in vivo* gamma amplitudes and with cognitive functions of the test animals (Lu et al., 2011). K_Ca_ channels open by an increase of intracellular calcium concentration and effectively control the firing of neurons by pulling the membrane potential to the reversal potential of potassium (Pedarzani and Stocker, 2008; Adelman et al., 2012; Contet et al., 2016). The present study shows that selective blockade of K_Ca_2 channels effectively increases the power of CA3 gamma oscillations evoked by cholinergic stimulation without noteworthy changes in the peak frequency. Since none of the selective activators and blockers of K_Ca_1.1 and K_Ca_3.1 channels affected gamma network activity it can be concluded that among all K_Ca_ channels only the K_Ca_2 channels had a gamma modulatory effect. K_Ca_2 channels are expressed on CA3 pyramidal cells (Sailer et al., 2002) and are besides KCNQ2/3 channels the only K_Ca_ channel subtype that is involved in AHPs of medium duration (mAHP) in these neurons (Storm, 1989; Sailer et al., 2002). Although K_Ca_3.1 channel proteins were found in the hippocampus (Turner et al., 2015), transcriptome and electrophysiological data suggest that they are not expressed in hippocampal cells and are not involved in the AHPs of pyramidal cells (Zeisel et al., 2015; Wang et al., 2016). The expression of K_Ca_1.1 channels in the hippocampus is ambiguous. While the channel was not found on somata and dendrites of pyramidal cells (Hu et al., 2001; Misonou et al., 2006; Sailer et al., 2006) it was shown to be involved in the repolarization of APs (Hu et al., 2001). Although the channel was found presynaptically on pyramidal cells (Misonou et al., 2006) and is suggested to decrease neurotransmitter release (Contet et al., 2016), axon terminal APs were not affected by channel blockers (Hu et al., 2001). Fast-spiking interneurons, involved in gamma oscillations, do not express K_Ca_1.1 channels (Erisir et al., 1999; Casale et al., 2015). Additionally, they are the only type of K_Ca_ channels which need membrane depolarization for opening and they have a lower Ca^2+^ activation affinity compared to K_Ca_2 and K_Ca_3.1 channels (Contet et al., 2016). Thus, for their opening K_Ca_1.1 channels need a coincident membrane depolarization and a relatively strong intracellular Ca^2+^ influx very close (ca. 10–50 nm; Contet et al., 2016) to the channels, a condition, which might not be given during gamma oscillations. Additionally, whether they inhibit or excite network activity (and decrease or increase neurotransmitter release) depends on the specific β subunits of the channel which have different kinetics and thus determine whether the channel speeds up the repolarization phase of the AP (β2; increasing the firing rate) or take part in the AHP (β4: decreasing firing; Contet et al., 2016). Taken together, K_Ca_1.1 channels seem to take part in the stabilization of network activity by getting activated by cumulated higher intracellular Ca^2+^ concentrations, which might explain their role in pathological processes in the brain such as epilepsy (Zhu et al., 2018). Our data, however, suggest that they don’t play a role in physiological network activity such as gamma oscillations.

Blockers of K_Ca_2 channels were shown to improve learning (Deschaux et al., 1997; Deschaux and Bizot, 2005; Kushwah et al., 2018), especially hippocampus-dependent spatial memory (Ikonen and Riekkinen, 1999; Stackman et al., 2002) and contextual fear memory (Vick IV et al., 2010). In contrast, overexpression of the K_Ca_2.2 channel severely impaired hippocampal-dependent spatial learning and memory (Hammond et al., 2006). Our results underscore these behavioral studies and suggest that K_Ca_2 channels might influence learning and memory by their role in gamma oscillations.

The voltage-dependent Kv7.2/3 (KCNQ2/3) potassium channels open already close to the resting membrane potential and determine herewith effectively the excitability and output of neurons (Greene and Hoshi, 2017). They are expressed in hippocampal pyramidal cells, generate mAHP (Storm, 1989), and are inhibited by activation of muscarinic M1 and M3 receptors (Brown and Adams, 1980). Therefore, Kv7.2/3 channels might be involved in gamma oscillations evoked by cholinergic stimulation. Our results indicate that blockade of the channels by XE991 effectively increases cholinergically induced gamma oscillations while the activator ICA110381 concentration-dependently attenuates them, suggesting that the inhibition of the M-type current is involved in the generation or maintenance of cholinergic gamma oscillations. However, although XE991 was able to further potentiate fully developed gamma oscillations it was not able to evoke synchronized activity when applied alone and we did not measure a significant increase of neuronal activity in the low gamma frequency range. This finding indicates that although the blockade of KCNQ2/3 channels is involved in the maintenance of cholinergically induced gamma oscillations, other muscarinic or nicotinic mechanisms must also be involved such as inhibition of K_Ca_2 channels (Buchanan et al., 2010) or activating a non-selective, presumably TRPC-based cation conductance (Yan et al., 2009). KCNQ channels seem not to affect the frequency since a decrease of the peak frequency was only observed when gamma oscillations were strongly or completely inhibited. Similar to our findings, KCNQ2/3 channel activators abolished or inhibited also kainate- or histamine-induced gamma oscillations (Boehlen et al., 2009, 2013; Andersson et al., 2017) indicating that KCNQ2/3 channels are not only involved in cholinergically evoked oscillations. Interestingly, XE991 was found to reduce the power of kainate-induced oscillations probably by loss of the periodicity of the increased firing of pyramidal cells (Boehlen et al., 2009; Leão et al., 2009) suggesting that the effect of the KCNQ2/3 blockers might depend on other channels affecting intrinsic excitability. In behavioral studies, inhibition of the M-current was shown to enhance learning and memory (Fontán-Lozano et al., 2011) and its suppression to be critical for memory consolidation (Kosenko et al., 2020), although the involvement of the KCNQ2/3 channels in memory is controversially discussed (Peters et al., 2005; Cavaliere et al., 2013; Li et al., 2014). Our results show that blockade of the M-current increases the power of hippocampal gamma oscillations induced by acetylcholine and suggest that this network mechanism might underlie the cognitive enhancing effects of KCNQ2/3 channel inhibitors.

The present study is to our knowledge the first that investigated the involvement of T-type calcium channels in gamma oscillations. T-type channels are low-voltage-activated channels that open near the foot of an action potential and play an important role in the generation of the afterdepolarizing potential and burst firing in CA3 pyramidal neurons, resulting in increased excitability of the cells (Xu and Clancy, 2008). Thus, the opening of these channels during cholinergic stimulation could be involved in the generation or maintenance of gamma oscillations. However, an enhanced calcium influx through an exogenous opening of T-type calcium channels, as seen after the application of SAK3, might activate K_Ca_ channels terminating the excitability of cells (Xu and Clancy, 2008) within the gamma circuit and decrease the power. Our results also show that a blockade of T-type channels increases the peak frequency of oscillations. The frequency of gamma oscillations can be modulated (increased) by the following mechanisms (Bartos et al., 2002; Jadi and Sejnowski, 2014): (a) shorter perisomatic inhibitory synaptic inputs; (b) reduced conduction delay in the gamma circuit; (c) decreased total inhibition on neurons of the circuit; and (d) reduced spacing between neurons (smaller network). Hippocampal interneurons express T-type channels more prominently compared to the cortex (McKay et al., 2006; Vinet and Sík, 2006; Aguado et al., 2016). Additionally, the downstream effect of T-type channels within individual cells might depend on the cellular and sub-cellular expression and activation state of K_Ca_ channels. Considering this, we can speculate that a blockade of T-type channels might affect interneurons stronger than pyramidal cells generating a decreased global inhibition of neurons within the network and an increase of the frequency.

Initially, low-voltage-activated T-type calcium channels were implicated in the synchronization of the thalamocortical circuit during sleep, absence seizures, and in peripheral and central pain transmission (Cheong and Shin, 2013). Later results, however, lead to the assumption that the channels might also be involved in higher cognitive functions, and both blockers and positive modulators were demonstrated to have pro-cognitive effects. Z944, an antagonist of the T-type channels, not only decreased seizure severity in a genetic rat model of absence epilepsy (Tringham et al., 2012) but also reversed learning and memory impairments (Marks et al., 2016, 2020). Similarly, the T-type channel enhancer SAK3 prevented cognitive impairment in different disease states and models such as hypothyroidism, ischemia, and AD models (Yabuki et al., 2017; Husain et al., 2018; Yuan et al., 2021) and showed beneficial effects in an animal model of intellectual disability syndrome (Dhanalakshmi et al., 2021). In addition, further studies suggest that enhancing these channels might have a pro-cognitive potential. Age-related downregulation of the channel mediated amyloid beta production (Rice et al., 2014) and SAK3 inhibited this in amyloid precursor protein transgenic animals (Xu et al., 2018). Moreover, T-type channel (Ca_v_3.2) lacking mice were shown to have impaired memory (Gangarossa et al., 2014). Our results demonstrating that T-type channels participate in gamma oscillations are in agreement with these studies and suggest that modulation of gamma oscillations might represent at least one working mechanism of how pharmacological manipulation of T-type channels alters higher cognitive tasks. However, since both the used blockers and enhancers inhibited hippocampal gamma oscillations, our data cannot help to answer the question whether a blockade or an opening of the channel is the preferential therapeutic strategy. Further studies are needed to assess the role of T-Type channels in synchronized network activity.

In conclusion, the present study investigated for the first time the role of K_Ca_ and T-type calcium channels in cholinergically evoked gamma oscillations. The presented data demonstrate that K_Ca_2 and T-type calcium channels are strongly involved in the modulation of gamma network activity and represent, besides KCNQ2/3 channels, effective pharmacological or genetic targets to increase the power of hippocampal gamma oscillations. Since gamma oscillations are associated with learning and memory, their rare positive modulators represent potential agents to treat cognitive impairment in different disease states.

## Data Availability Statement

The raw data supporting the conclusions of this article will be made available by the authors on request, without undue reservation.

## Ethics Statement

The animal study was reviewed and approved by Landesamt für Gesundheit und Soziales (LAGeSo) Berlin, Turmstraße 21, 10559 Berlin, Germany.

## Author Contributions

AK and ET performed the LFP experiments and analyzed the results. FW conducted the patch clamp recordings and analyzed the data. ZG designed the experiments, analyzed and interpreted the data. AK, FW, and ZG drafted the manuscript. All authors contributed to the article and approved the submitted version.

## Conflict of Interest

The authors declare that the research was conducted in the absence of any commercial or financial relationships that could be construed as a potential conflict of interest.

## Publisher’s Note

All claims expressed in this article are solely those of the authors and do not necessarily represent those of their affiliated organizations, or those of the publisher, the editors and the reviewers. Any product that may be evaluated in this article, or claim that may be made by its manufacturer, is not guaranteed or endorsed by the publisher.
